# Supplementary material for: Development of a standardized consensus lexicon for terms related to micronutrient programs
Source: PLoS One. 2024 Aug 30;19(8):e0308230. doi: 10.1371/journal.pone.0308230 (PMC11364245; doi:10.1371/journal.pone.0308230)
Supplement: S1 File — (DOCX) [file pone.0308230.s001.docx]

**DEVELOPMENT OF A STANDARDIZED CONSENSUS LEXICON FOR TERMS RELATED TO MICRONUTRIENT PROGRAMS**

**Supplementary material (1)**

**List of references for terms search in Lexicon**

1. ACT No. XXVII of Balochistan provincial assembly secretariat: The Balochistan Food Authority Act (2021).
2. Allen, L. H., A. L. Carriquiry and S. P. Murphy (2020). "Perspective: proposed harmonized nutrient reference values for populations." Advances in Nutrition 11(3): 469-483.
3. Allen, The SAGE Encylopedia of Communication Research Methods, 2017
4. Article 3(18) of the Regulation (EC) No 178/2002 of the European Parliament and of the Council
5. Balochistan provincial assembly secretariat: The Balochistan Food Authority Act, (2014).
6. Black’s Law Dictionary, Eleventh Edition, (2019).
7. European Food Safety Authority, Food contact materials
8. FAO (2021). Minimum dietary diversity for women: An updated guide to measurement-from collection to action, Food and Agriculture Organization of the United Nations.
9. FAO and WHO (2021): Guidelines on Nutrition Labelling (CXG 2-1985)
10. FAO Food Safety and Standards (Advertising and Claims) Regulations (2018)
11. FAO, WHO (2011): General principles of food hygiene CXC 1-1969
12. FAO, WHO (2011): General principles of food hygiene CXC 1-1970
13. FAO, WHO (2011): General Principles of Food Hygiene, (CXC 1-1969).
14. FAO, WHO (2018): General standard for the labelling of prepackaged foods: CXS 1-1985
15. FAO, WHO: General Principles of Food Hygiene, (CXC 1-1969).
16. Food Safety and Standards (Advertising and Claims) Regulations (2018)
17. Food Safety and Standards (Imports) Regulations, (2017).
18. Food Safety and Standards (Labelling and Display) Regulations (2020)
19. Food Safety and Standards (Packaging) Regulations (2018)
20. Global Alliance for Improved Nutrition (GAIN): https://www.gainhealth.org/
21. Global fortification data exchange (GFDx) indicator definitions and data inclusion criteria, 2019.
22. Global Fortification Data Exchange (GFDx): https://fortificationdata.org/
23. Global Fortification Data Exchange (GFDx): Methodology, Definitions of GFDx indicators
24. Hess, S. Y., A. C. McLain, E. A. Frongillo, A. Afshin, N. J. Kassebaum, S. J. M. Osendarp, R. Atkin, R. Rawat and K. H. Brown (2021). "Challenges for Estimating the Global Prevalence of Micronutrient Deficiencies and Related Disease Burden: A Case Study of the Global Burden of Disease Study." Current Developments in Nutrition 5(12).
25. https://en.wikipedia.org/wiki/Data_library
26. Institute of Medicine (2000). Dietary Reference Intakes: Applications in Dietary Assessment, The National Academies Press.
27. Iodine Global Network: https://ign.org/
28. King’s College London, OpeN-Global: https://www.kcl.ac.uk/open-global
29. Micronutrient Forum: https://micronutrientforum.org/
30. Office of Research Integrity: Responsible conduct in data management
31. Olson, R., B. Gavin-Smith, C. Ferraboschi and K. Kraemer (2021). "Food Fortification: The Advantages, Disadvantages and Lessons from Sight and Life Programs." Nutrients 13(4): 1118.
32. Open Data Watch, https://opendatawatch.com/ : access on 12 April
33. OpeN-Global, King’s College London (https://www.kcl.ac.uk/open-global )
34. Oracle Cloud Free Tier: What is database?
35. Osendarp, S. J., H. Martinez, G. S. Garrett, L. M. Neufeld, L. M. De-Regil, M. Vossenaar and I. Darnton-Hill (2018). "Large-scale food fortification and biofortification in low- and middle-income countries: a review of programs, trends, challenges, and evidence gaps." Food and nutrition bulletin 39(2): 315-331.
36. Packaged Food Labelling Act, (2017).
37. PATH and GAIN (2015). Rice fortification toolkit: technical manual: https://www.gainhealth.org/resources/reports-and-publications/rice-fortification-toolkit-technical-manual
38. PATH and GAIN (2015). Rice fortification toolkit: technical manual: https://www.gainhealth.org/resources/reports-and-publications/rice-fortification-toolkit-technical-manual
39. Punjab Pure Food Regulations (2018)
40. Regulation on Food Safety (Chemical Contamination, toxin, and harmful residue), (2017).
41. Science Direct: Target population: https://www.sciencedirect.com/topics/engineering/target-population
42. Science Direct (2015) (https://www.sciencedirect.com/topics/biochemistry-genetics-and-molecular-biology/essential-nutrient)
43. Science Direct: Dairy products: https://www.sciencedirect.com/topics/agricultural-and-biological-sciences/dairy-product
44. Sight and Life supplement covering the Scaling up Rice Fortification in Asia meeting in Bangkok (2014).
45. Sindh Compulsory Iodization of Salt Act (2013): Provincial assembly of Sindh notification Karachi.
46. Tableau, Guide To Data Cleaning: Definition, Benefits, Components, And How To Clean Your Data: access at https://www.tableau.com/learn/articles/what-is-data-cleaning on 12 April 2023
47. The Regulation (EC) No 178/2002 of the European Parliament and of the Council
48. The Regulation (EC) No 178/2002 of the European Parliament and of the Council
49. The Right to Food and Food Sovereignty Act, 2075 (2018)
50. The United Nations: Peace, dignity and equality on a healthy planet
51. UNICEF Nutrition: https://www.unicef.org/nutrition
52. UNICEF: The Convention on the Rights of the Child, The children’s version: https://www.unicef.org/child-rights-convention/convention-text-childrens-version
53. USAID (2022). Large-Scale Food Fortification Programming Guide: Supporting Food Fortification at a Country Level and on a Global Scale: https://www.advancingnutrition.org/resources/usaid-large-scale-food-fortification-programming-guide-supporting-food-fortification
54. WHO (2018). "Guideline: fortification of rice with vitamins and minerals as a public health strategy."
55. WHO (2021). "Monitoring flour fortification to maximize health benefits: a manual for millers, regulators, and programme managers."
56. WHO (2021). National nutrition information systems – the fundamental series, modules 1–5, New York: United Nations Children’s Fund (UNICEF) and the World Health Organization.
57. WHO and FAO (2006). Guidelines on food fortification with micronutrients: https://www.who.int/publications/i/item/9241594012
58. WHO, Key facts on food additives: https://www.who.int/news-room/fact-sheets/detail/food-additives#:~:text=Key%20facts,modify%20sensory%20properties%20of%20food.
59. WHO, Adolescent health: https://www.who.int/health-topics/adolescent-health#tab=tab_1
60. Wikipedia. Data curation: https://en.wikipedia.org/wiki/Data_curation
61. Wikipedia. Data exchange: https://en.wikipedia.org/wiki/Data_exchange
62. World Food Programme (WFP): https://www.wfp.org/
