# Supplementary material for: Development of a standardized consensus lexicon for terms related to micronutrient programs
Source: PLoS One. 2024 Aug 30;19(8):e0308230. doi: 10.1371/journal.pone.0308230 (PMC11364245; doi:10.1371/journal.pone.0308230)
Supplement: S2 File — (DOCX) [file pone.0308230.s002.docx]

**DEVELOPMENT OF A STANDARDIZED CONSENSUS LEXICON FOR TERMS RELATED TO MICRONUTRIENT PROGRAMS**

**Supplementary material 2**

**List of organizations of the experts who contributed to the review of Lexicon terms**

| **Sr.** | **Name of organizations/ institutions** |
| --- | --- |
|  | Bill and Melinda Gates Foundation |
|  | Department of Community Medicine and School of Public Health, Postgraduate Institute of Medical Education and Research (PGIMER), Chandigarh, India |
|  | Deutsche Gesellschaft für Internationale Zusammenarbeit (GIZ) |
|  | Faculty of Medicine, Sofia University, Bulgaria |
|  | Food and Agriculture Organization (FAO) |
|  | Global Alliance for Improved Nutrition (GAIN) |
|  | Institute of Nutrition, Mahidol University |
|  | International Zinc Nutrition Consultative Group (IZINCG) |
|  | Iodine Global Network (IGN) |
|  | Johns Hopkins University |
|  | London School of Hygiene and Tropical Medicine |
|  | Micronutrient Data Innovation Alliance (DInA) |
|  | Micronutrient Forum |
|  | Nutrition International |
|  | Program for Appropriate Technology in Health (PATH) |
|  | Shahabuddin Medical College Hospital |
|  | United Nations International Children's Emergency Fund (UNICEF) |
|  | University of California, Davis |
|  | University of Chile |
|  | University of Otago, New Zealand |
|  | University of the Philippines Los Baños |
|  | United Nations World Food Programme (WFP) |
|  | World Health Organization (WHO) |
