# Supplementary material for: Development of a standardized consensus lexicon for terms related to micronutrient programs
Source: PLoS One. 2024 Aug 30;19(8):e0308230. doi: 10.1371/journal.pone.0308230 (PMC11364245; doi:10.1371/journal.pone.0308230)
Supplement: Attachment — Submitted filename: Response to Reviewers_20240715.docx [file pone.0308230.s004.docx]

**Development of a standardized consensus lexicon for terms related to micronutrient programs**

We all thank the editors and reviewers for their time to review the abovementioned manuscript (ID: PONE-D-24-04315) and provide valuable feedback for improvement. Please see below our point-by-point response to all comments in blue. The revisions are made with track changes, and we also submitted a cleaned version of the revised manuscript. The line numbers correspond to the marked version. We hope that you will find the revisions to the manuscript thorough and satisfactory. We look forward to receiving a further response from you.

**Reviewer #1**:

This manuscript provides a valuable resource in standardizing terminology across micronutrient programs. Below are my key points and recommendations for improvement.

**Methodology**

The methodology section is well-explained but could emphasize the criteria for term selection and the process of consensus-building among experts. This would strengthen the rigor and transparency of the lexicon development process.

We have added the description that the selection and categorization of terms was based on different approaches of micronutrient interventions (fortification, supplementation, dietary diversification) and the phases of program cycles, i.e., assessment, planning, implementation, monitoring and evaluation (lines 30-32, 111-113, and 115-116).

A key weakness in the methodology is the apparent absence of food manufacturers/processors (of fortified food) and food regulators and/or food safety experts in the list of experts who reviewed the Lexicon of terms. These stakeholders often rely on precise definitions of terms to develop regulatory standards and to comply with these standards.

We have circulated the online survey forms to solicit the opinions to a larger group of experts (more than 140 in number) including experts in food legislation and food safety. There were experts in policy development, food industry regulations, and food safety among the 39 experts who have responded to the online survey. Legal terms were also added in the lexicon in consultation with a legal expert, who contributed as coauthor to this manuscript. We have added further details to the methods section (lines 166-167, 180) and results section (lines 199-201). Despite the above-described efforts, we were not able to obtain feedback from food manufacturers in the consensus building process of the current lexicon. We have added this in the discussion (lines 234-236 and lines 247-249) as potential weakness and for consideration when adding new terms to the lexicon.

**Results and Discussion**

The results are clearly presented, showing the extensive work done to compile and refine the lexicon. The discussion highlights the lexicon's potential impact but should address the low response rate in the expert surveys more critically, discussing its implications on the representativeness and global consensus of the definitions.

Thank you for this comment. The discussion on the low response rate of expert survey has been edited as suggested (lines 231-236).

Lines 174-185: The presentation of data is clear, but it could be enhanced by discussing the implications of these findings. For instance, what does the categorization of terms indicate about the focus areas in micronutrient programs? Also, consider presenting the data in a table for easier comprehension and reference.

As suggested, we have added **Table 1** (line 140) to provide an overview of the categories and sub-categories that guided the search for terms. We also clarified that rationale for considering these terms as follows on lines 30-32, 111-113, and 115-116: “To provide a clear framework for program management, and to ensure all aspects of the program are systematically addressed, the terms were categorized according to the programmatic phase,…”

Lines 195-225: The discussion effectively highlights the lexicon's importance and potential impact. However, the challenges and limitations mentioned could be better framed as opportunities for future engagement and development. For example, the low response rate could be addressed by discussing strategies to increase participation and representation in future updates.

We have added this to the discussion lines 231-236 and 247-249.

Line 216-219: The commitment to maintaining the lexicon as a living document is commendable. Expanding on how this will be achieved, including the process for incorporating new terms and updating definitions, would be beneficial.

The discussions on the process of incorporating new terms and updating definitions, along with a plan to increase participation and representation in future updates are added on lines 242-249.

**General Recommendations**

Provide a clearer rationale for categorizing terms and how these categories aid the lexicon’s usability for different stakeholders.

As mentioned above, we have added the description on lines 30-32, 111-113, and 115-116 and adding Table 1.

Emphasize the impact of the lexicon in the discussion, particularly how it can enhance communication and decision-making in micronutrient programs. Discuss the real-world application of the lexicon, such as how it has been used in policy-making, program design, or academic research, to demonstrate its practical value.

The potential implications of the lexicon has been addressed in lines 250-252.

The manuscript represents a significant effort in standardizing terminology in the field of nutrition and has the potential to be a vital resource for stakeholders in micronutrient programs. With the recommended improvements, I believe it will make a substantial contribution to the literature. However, as mentioned above, the absence of manufacturers of fortified foods and food regulators and/or food safety experts concerns me.

Thank you very much again for your valuable comments and suggestions to improve our manuscript. The critical discussion and future plan to include food manufacturers when adding new terms was described on lines 234-236 and lines 246-252.

**Reviewer #2**:

This is a very useful effort to standardize the lexicon and terminology used by different stakeholders working to address micronutrient deficiencies. The methodology used is sound and I recommend the publication of the paper without any modifications.

Thank you very much for your review and recommendation for publication.
